# Sensitivity of *Paucilactobacillus wasatchensis* strains to nisin

**DOI:** 10.3168/jdsc.2025-0887

**Published:** 2025-10-18

**Authors:** Kate Sorensen, Niharika Mishra, Taylor S. Oberg

**Affiliations:** Department of Nutrition, Dietetics and Food Sciences, Utah State University, Logan, UT 84322

## Abstract

*Paucilactobacillus wasatchensis* is a nonstarter lactic acid bacterium that has been isolated throughout the United States and is associated with late blowing gas defect (LBD), a continuing problem in the cheese industry, which results in downgraded product and economic loss. Although the route of *Pa. wasatchensis* contamination in milk destined for cheesemaking remains inconclusive, the addition of nisin or the application of nisin-producing starter cultures in Cheddar production may mitigate LBD by inhibiting *Pa. wasatchensis.* Nisin sensitivity has not yet been evaluated for *Pa. wasatchensis* strains. The goal of the current study was to evaluate 8 strains of *Pa. wasatchensis* for nisin sensitivity using the agar well diffusion method. Nisin inhibition was evaluated across a range from 5 to 100 IU/mL. To compare strain sensitivity, the lowest nisin concentration that displayed inhibition for all strains was selected (30 IU/mL), and the corresponding inhibition zones were statistically analyzed by ANOVA with a Tukey–Kramer post hoc test. The MIC for each strain were also estimated. Each *Pa. wasatchensis* strain showed inhibition within the tested range of nisin concentration. Results from the Tukey–Kramer post hoc test showed some strain differences in nisin sensitivity, but these were not correlated with region of isolation. All strains showed inhibition at 30 IU/mL nisin, which indicates that *Pa. wasatchensis* is highly sensitive to nisin. This supports addition of nisin, or the application of nisin protective cultures, as a potential effective strategy in mitigating LBD caused by *Pa. wasatchensis*.

## Highlights

• All Pa. wasatchensis strains evaluated showed inhibition at 30 IU/mL nisin.
• Statistical analysis showed differences in nisin sensitivity between some *Pa. wasatchensis* strains.
• Overall, *Pa. wasatchensis* is highly sensitive to nisin.
• Results support that nisin use in cheesemaking may effectively inhibit *Pa. wasatchensis*.

## Graphical Abstract

**Figure:**
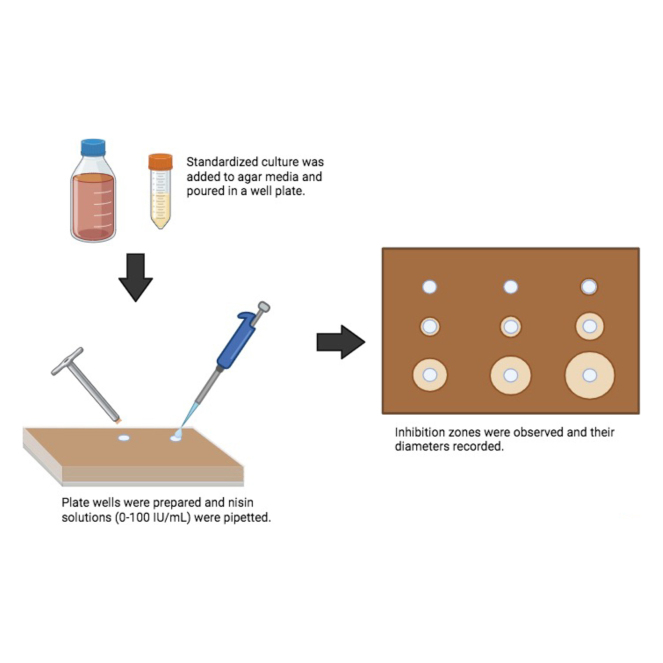
**Summary:***Paucilactobacillus wasatchensis* is a lactic acid bacterium associated with late blowing gas defect (LBD) in Cheddar cheese throughout the United States, which results in downgrading of product and economic loss for the cheese industry. Determining strategies to mitigate LBD caused by *Pa. wasatchensis* is of major interest. The addition of nisin, a bacteriocin that is commonly applied in dairy foods to mitigate spoilage, or the application of nisin-producing cultures are promising strategies. Using such strategies to reduce LBD caused by *Pa. wasatchensis* will alleviate economic losses while maintaining consumer acceptance, both of which are vital to the cheese industry.

Cheddar cheese continues to be the most popular natural cheese consumed in the United States, due in part the flavor and textural properties developed during ripening (Racette et al., 2024). During manufacture, the primary starter lactic acid bacteria (**SLAB**) cultures drive acid development during the cheese make and contribute to the flavor characteristics of young curd. As the cheese ripens, the SLAB organisms begin to lose viability and undergo autolysis, and the population shifts to thermoduric bacteria that are part of the natural microbiota of the milk or processing environment, termed nonstarter lactic acid bacteria (**NSLAB**; Beresford and Williams, 2004). Although some NSLAB are considered adventitious contaminants and increase flavor development of aging cheese, others have been shown to cause defects in Cheddar cheese, such as textural defects and production of unwanted CO_2_ during the aging process (Laleye et al., 1987; Khalid and Marth, 1990; Mullan, 2000). This late blowing gas defect (**LBD**) in Cheddar cheese causes slits and cracks within the cheese block and bloated packaging, ultimately resulting in downgrading of the product, with associated economic loss to the manufacturer (Elliott et al., 1981).

One recently characterized heterofermentative NSLAB is *Paucilactobacillus wasatchensis*, an organism isolated from aged Cheddar cheese with LBD in several regions throughout the United States (Oberg et al., 2016; Culumber et al., 2017). Previous research has shown that Cheddar cheese purposefully inoculated with *Pa. wasatchensis* cells resulted in a significant increase of gas production and gassy cheese defect, indicating the organism as a potential model NSLAB for LBD (Ortakci et al., 2015b,c; McMahon et al., 2022). Studies have also identified metabolic mechanisms for *Pa. wasatchensis* gas production and determined that specific 6-carbon sugars or sugar derivatives, including galactose and sodium gluconate, can be metabolized by *Pa. wasatchensis* to produce CO_2_ (Ortakci et al., 2015a; Oberg et al., 2021).

Although a great deal of research has been conducted on this organism's metabolic capabilities to produce CO_2_ in cheese, the route of contamination is currently undecided. Initial whole-genome sequence analysis revealed that *Pa. wasatchensis* shares high DNA homology to lactobacilli species isolated from agricultural environmental sources such as timothy grass silage, cow dung, soil, and compost (Oberg et al., 2016). A recent study was able to detect *Pa. wasatchensis* in raw milk and corn silage samples using nonculture PCR-based methods (Chiu et al., 2023). An organism from such agricultural sources has the potential to contaminate milk destined for cheese production via environmental contamination (Martley and Crow, 1993). Recent studies examining the effects of heat treatment on *Pa. wasatchensis* have yielded inconsistent results regarding the organism's ability to survive pasteurization (McMahon et al., 2020; Lampien et al., 2023), with some reports suggesting post-pasteurization contamination or survival within biofilms, whereas others provide evidence supporting heat tolerance during pasteurization (Ortakci et al., 2015a; Chiu et al., 2023). Despite research in this area, the exact mechanism of milk contamination has not been distinctly pinpointed, and an effective means of preventing *Pa. wasatchensis* contamination in milk used for cheese production has not been determined.

Because the mode of *Pa. wasatchensis* contamination has not yet been established, it has become necessary to determine mitigations that lessen LBD development during ripening. Studies have identified risk factors that might increase LBD from *Pa. wasatchensis*, including elevated temperature during aging or incorporation of sodium gluconate (Ortakci et al., 2015b; McMahon et al., 2022). However, any mitigation to decrease LBD caused by *Pa. wasatchensis*, or similar NSLAB, should not alter the cheese make or interfere with flavor development during aging of the cheese product. The use of bioprotective cultures added during the cheese make has shown potential to decrease or eliminate LBD, with research indicating that the use of cultures which ferment galactose but not lactose will reduce CO_2_ formation when grown together with *Pa. wasatchensis* in vitro (Green et al., 2021). When these cultures were then tested in Cheddar cheese inoculated with *Pa. wasatchensis*, they showed a reduction in LBD. Results showed that gas production was still sporadic, and reduction was dependent on the bioprotective culture and the heterofermentative NSLAB that produced the LBD defect (R. Crompton, D. J. McMahon, and T. S. Oberg, Utah State University, Logan, UT; unpublished data).

The use of nisin-producing starter bacteria to control gas production in cheese was suggested by Mullan (2000) as a potential form of biological control. Nisin is a class I bacteriocin shown to have antimicrobial activity against a wide range of gram-positive organisms while also being considered a natural, toxicologically safe biopreservative (D'Amato and Sinigaglia, 2010). Nisin has received Generally Recognized as Safe (GRAS) status and meets the increasing consumer demand for clean-label food (Anumudu et al., 2022; FDA, 2023). As nisin is stable and has increased solubility under acidic conditions, its application in fermented dairy is proven. The milk protein-nisin interactions in cheesemaking also ensure high retention of nisin within the cheese matrix (Ibarra-Sánchez et al., 2020). Recent studies have already investigated the incorporation of nisin, or nisin-producing cultures, to inhibit *Clostridium* spp. that are similarly associated with LBD in cheese (Ávila et al., 2020; Akbal and Öner, 2024). This suggests that nisin application may be a practical method of controlling LBD produced by *Pa. wasatchensis* as well.

Methods to directly determine nisin sensitivity of bacteria in vitro have varied in literature and include microtiter dilution (Rojo-Bezares et al., 2007), deferred inhibition testing (Carl et al., 2004), agar diffusion assay (Ogden and Tubb, 1985; Radler, 1990), and streaking on agar plates containing a range of nisin concentrations (Carl et al., 2004). The benefits of each method vary, but the agar well diffusion method is a widely used conventional method for determination of sensitivity (Pongtharangkul and Demirci, 2004). This assay is based on the measurement of zones of inhibition in agar that is seeded with an organism of interest, and is commonly used to determine the concentration of nisin by using an indicator organism with known sensitivity (i.e., *Micrococcus luteus*; Wolf and Gibbons, 1996; Chandrasekar et al., 2015). This method has also been employed as a conventional means of determining antimicrobial activity for a wide variety of substances against a diverse array of organisms (Magaldi et al., 2004; Nalawade et al., 2016).

As there is no published data on nisin sensitivity for any *Pa. wasatchensis* strains, the purpose of this study was to establish baseline nisin inhibition characteristics of all available strains of this organism. To accomplish this, an agar diffusion bioassay (Pongtharangkul and Demirci, 2004) was used to test 8 *Pa. wasatchensis* strains for nisin sensitivity. The results allowed for comparison of nisin sensitivity among strains to determine any correlation between region of isolation and nisin sensitivity and to provide information on the potential efficacy of addition of nisin or nisin-producing protective cultures to cheese to mitigate LBD associated with this organism.

Strains selected for testing were obtained from the Utah State University Food Science culture collection and are listed with identification codes in Table 1. All cultures grown from freezer stocks were inoculated into de Man, Rogosa, and Sharpe (**MRS**) broth (Difco, Becton, Dickinson and Company, Sparks, MD) supplemented with 1% ribose (**MRSr**; Sigma Aldrich, St. Louis, MO) and incubated anaerobically (BD Gaspak, Becton, Dickinson and Company, Sparks, MD) for 72 h at 25°C. A quadrant streak plate was prepared for each broth culture on MRSr agar and incubated as described above. For nisin inhibition testing, cultures were prepared for inoculation by selecting an isolated colony from the MRSr agar plates and inoculating into 25 mL of MRS broth supplemented with 1% ribose and 1% galactose (**MRSrg**; Acros Organics, Fair Lawn, NJ) and incubated for 72 h at 25°C. The cultures were then diluted to an optical density at 600 nm (**OD_600_**) of 1.2 ± 0.05 (acceptable range) using the same broth type. The cell density was confirmed by standard serial plating on MRSr agar and incubated anaerobically at 25°C for 4 d.

**Table 1:** *Paucilactobacillus wasatchensis* strains used for nisin testing

Strain identification code	US region of isolation	Isolation cheese type
NW1	Northwest^1^	Aged Cheddar
NW2	Northwest^1^	Aged Cheddar
IM3	Intermountain^1^	Cheddar (flavored)
IM4	Intermountain^1^	Cheddar (flavored)
MW5	Midwest^1^	Dill Havarti
MW6	Midwest^1^	Monterey Jack
SK0033	Northeast^2^	Commercial cheese (no type identified)
WDC04	Intermountain^3^	Aged Cheddar

1 Culumber et al., 2017.

2 Data not published.

3 Oberg et al., 2016.

Nisin inhibition testing for each strain was conducted using an agar well diffusion method. To prepare the medium, MRSrg agar supplemented with 1% Tween-20 (Bio-Rad Laboratories, Hercules, CA) was prepared and autoclaved at 121°C for 15 min followed by tempering in a water bath to 48°C. After tempering, 5% of the prepared inoculum culture (OD_600_ = 1.2) was aseptically pipetted into the medium, which was stirred rapidly for 10 s using a stir plate; then 90 mL of the inoculated agar were immediately poured into cell culture plates (12.78 × 8.55 cm; Thermo Fisher, Rochester, NY). Additional plates were poured without the addition of any culture as negative controls. This was performed in duplicate (biological replicates) for each strain and control. The plates were allowed to set at room temperature. After solidifying, a metal cork-borer (7-mm diameter) was sterilized via ethanol solution and flaming and used to punch 9 holes evenly spaced into each agar plate following a template.

A 1,000 IU/mL stock solution of nisin from *Lactococcus lactis* (Sigma Aldrich, St. Louis, MO) was made in sterile 0.02 *N* HCl. Further dilutions with 0.02 *N* HCl were made to achieve concentrations (IU/mL) of 100, 60, 45, 30, 15, 10, and 5. For testing, 200 µL of each prepared nisin concentration was placed into 8 of the wells, and 200 µL of 0.02 *N* HCl solution was placed into the remaining well and designated as 0 IU/mL nisin. Plates were incubated upright anaerobically at 25°C for 72 h. Inhibition zones were measured as the diameter of a visible clearing around each well of nisin solution and reported in millimeters. The entire testing protocol was conducted in duplicate. Statistical analysis was conducted using JMP ver.18.2.1 (JMP Statistical Discovery LLC, Cary, NC).

Results obtained from enumeration of the prepared inoculum show final cell densities between replicates in a range of 7.1 to 8.9 log_10_ cfu/mL for all strains. Inhibition zones were apparent for all strains at 72 h of incubation, with each strain exhibiting inhibition zones within our tested range of 5 to 100 IU/mL. For comparison, mean inhibition zone measurements (mm) for all strain replicates were plotted against the corresponding nisin concentrations (IU/mL) to obtain inhibition curves (Figure 1). The slope of the inhibition curves varied by strain, with IM3, NW2, and SK0033 showing the steepest slopes (that is, larger inhibition zones as the nisin concentration increases), indicating greater sensitivity compared with the other strains. In contrast, NW1 and WDC04 showed more moderate slopes, and IM4 and MW6 demonstrated even more gradual responses, reflecting moderate sensitivity among these strains. Strain MW5 displayed the flattest slope, with only small incremental increases in inhibition zone size as nisin concentration increased, suggesting the lowest sensitivity to nisin among the strains tested.

**Figure 1:**
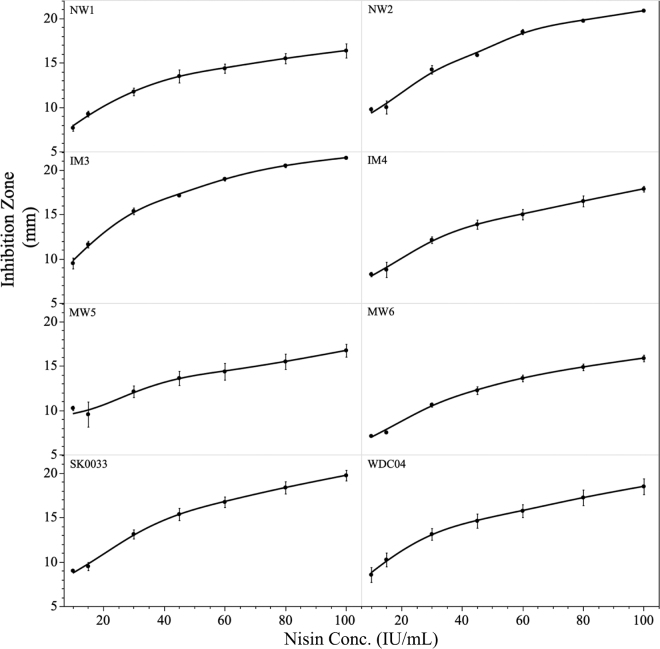
Inhibition zones (mm) across nisin concentrations (conc.; IU/mL), expressed as mean of all replicates ± SE for *Paucilactobacillus wasatchensis* strains. Strain identification codes are given in Table 1.

To better understand strain level differences in sensitivity of regional isolates of *Pa. wasatchensis*, the lowest nisin concentration that exhibited a measurable inhibition zone in all strains, 30 IU/mL, was selected to compare average inhibition zone measurements across strains. Differences between inhibition zones at this concentration were compared using a one-way ANOVA with a Tukey–Kramer honest significant difference (**HSD**) post hoc test. As shown in Figure 2, statistical differences were detected between specific strains, but no consistent pattern emerged when compared by geographical region. Strains IM3 and IM4, both Intermountain isolates, were statistically different from each other, but both were similar to the original Intermountain isolate, WDC04. The 2 Northwest isolates, NW1 and NW2, were statistically different from each other, whereas strains MW5 and MW6, both isolated in the Midwest, were statistically similar. The only strain isolated in the Northeastern region, SK0033, was statistically similar to all strains except MW6. Although statistically significant differences exist between strains, it is important to distinguish between statistical and biological significance when interpreting these results. Although relatively small differences in inhibition zones between strains may be statistically significant due to low variability among replicates, they are unlikely to reflect meaningful biological differences in sensitivity (Bennik et al., 1997; Carl et al., 2004).

**Figure 2:**
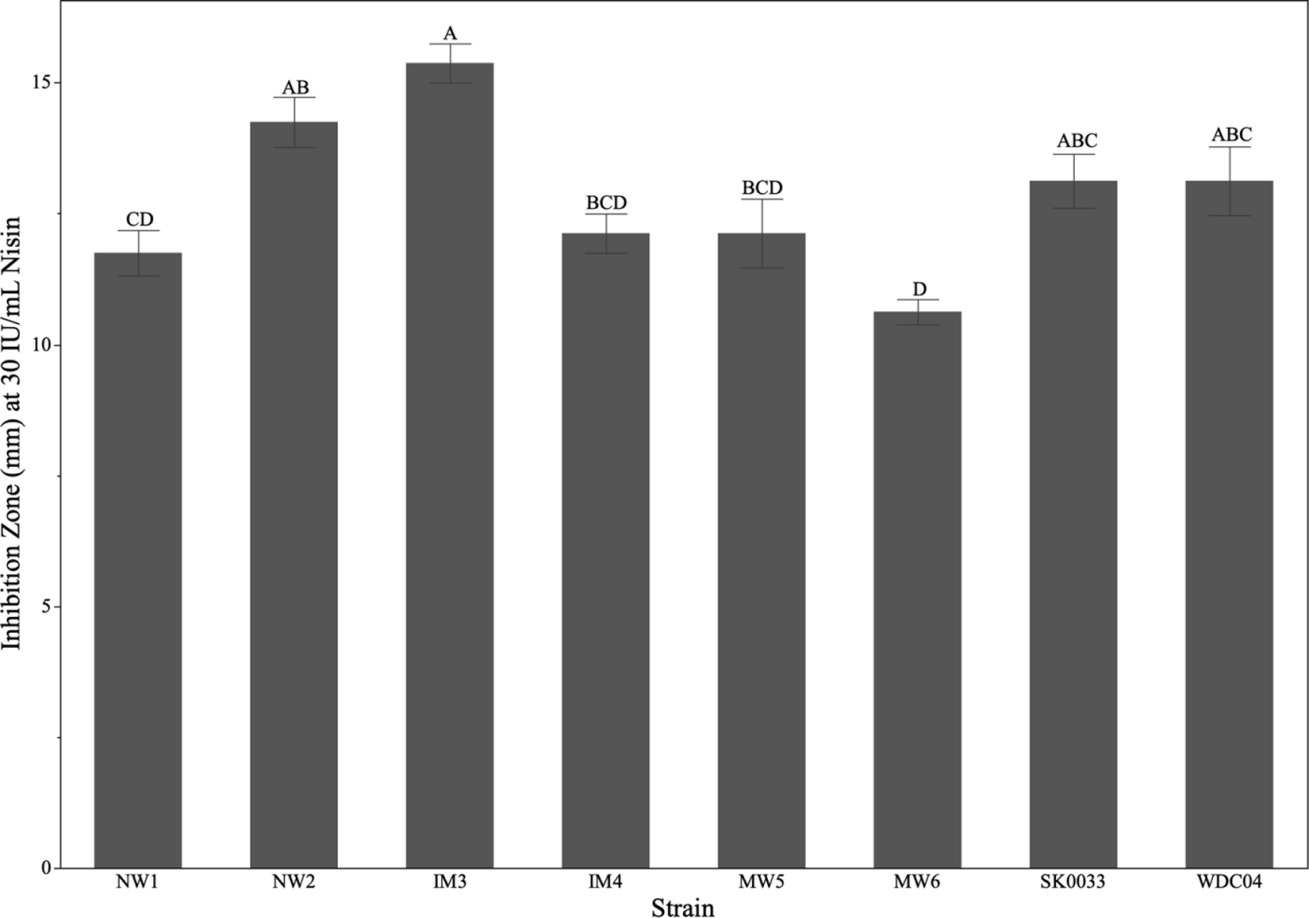
Inhibition zone measurements of 30 IU/mL nisin for *Paucilactobacillus wasatchensis* strains (identification codes given in Table 1), expressed as averages of all replicates ± SE. Statistically significant differences (one-way ANOVA with Tukey–Kramer HSD post hoc) are denoted by letters (A–D) above each strain. Strains with the same letter are not statistically significantly different.

Compared with previous studies that tested more widely spaced nisin concentrations (Radler, 1990; Carl et al., 2004), the present study examined a broader set of concentrations within a narrower range. For each replicate, MIC were estimated as the lowest nisin concentration that produced an observed zone of inhibition. Based on the data, the estimated MIC values (IU/mL) for *Pa. wasatchensis* strains were as follows: NW1 ≤10; NW2 ≤10 to 15; IM3 ≤10; IM4 ≤10 to 15; MW5 ≤10 to 15; MW6 ≤10 to 30; SK0031 ≤10; SK0033 ≤10 to 15; and WDC04 ≤10. Although broth assays, as used by Radler (1990), can provide more precise MIC values, the narrow ranges estimated here still offer meaningful insight into the nisin sensitivity of *Pa. wasatchensis* strains. The estimated MIC values for *Pa. wasatchensis* strains in this study are consistent with previous reports of nisin inhibition among lactic acid bacteria. For example, strains of *Lactiplantibacillus pentosus* and *Lactiplantibacillus plantarum* exhibited nisin MIC of 10 IU/mL on an agar diffusion assay (Carl et al., 2004). In broth assays, *Leuconostoc* strains were fully inhibited by 2 IU/mL nisin, and *Pediococcus* strains were inhibited at 4 to 8 IU/mL (Radler, 1990). The same study reported inhibition of *Levilactobacillus brevis* and *Lentilactobacillus hilgardii* at 32 IU/mL. In addition, 2 *Latilactobacillus sakei* strains had MIC values of 15.6 µg/mL (equivalent to 15.6 IU/mL), whereas another *L. sakei* strain and a *Latilactobacillus curvatus* strain had MIC values below 0.5 µg/mL (Chung and Hancock, 2000).

Prior research that has investigated the use of nisin in manufactured Cheddar cheese, either by using nisin-producing starter cultures or by intentionally adding nisin, found the resulting cheese to have nisin concentrations ranging from 400 to over 1,000 IU/g (Roberts et al., 1992; Zottola et al., 1994). Our results show that the lower reported concentration of nisin found in Cheddar cheese (i.e., 400 IU/mL) would be over 10 times the MIC concentration of *Pa. wasatchensis* strains. This suggests that incorporation of nisin-producing cultures in Cheddar manufacture would be an effective means to inhibit the growth of *Pa. wasatchensis* and potentially reduce risk of LBD associated specifically with this organism.

Results from this study demonstrate a high sensitivity of *Pa. wasatchensis* strains to the food-grade bacteriocin nisin. Slight differences in nisin sensitivity were observed between some strains but showed no correlation to the US region of strain isolation. Although statistical differences did exist between some strains, all strains showed complete inhibition at 30 IU/mL or less of nisin. This indicates that that the addition of nisin, or the application of nisin-producing protective cultures, in Cheddar cheese manufacture may be an effective mitigation strategy of LBD caused by *Pa. wasatchensis.*
